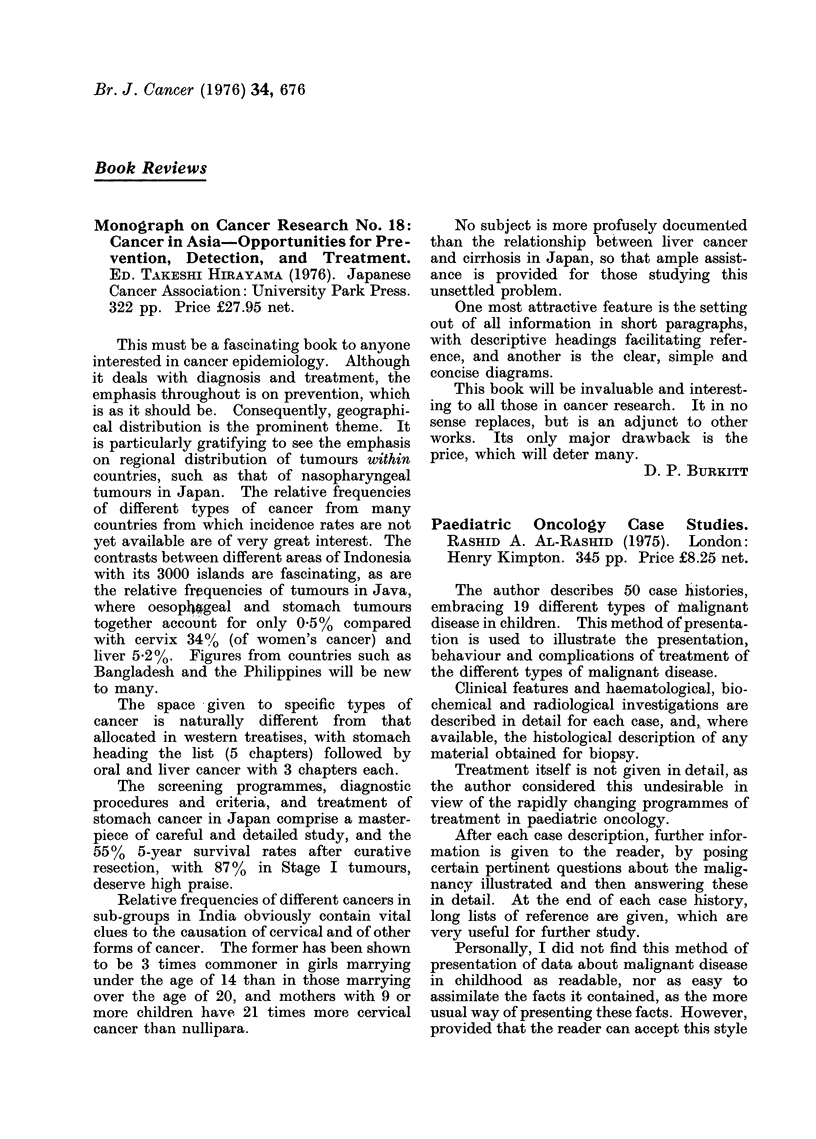# Monograph on Cancer Research No. 18: Cancer in Asia—Opportunities for Prevention, Detection, and Treatment

**Published:** 1976-12

**Authors:** D. P. Burkitt


*Br. J. Cancer* (1976) **34**, 676

### *Book Reviews*

**Monograph on Cancer Research No. 18: Cancer in Asia—Opportunities for Prevention, Detection, and Treatment.** ED. TAKESHI HIRAYAMA (1976). Japanese Cancer Association: University Park Press. 322 pp. Price £27.95 net.

This must be a fascinating book to anyone interested in cancer epidemiology. Although it deals with diagnosis and treatment, the emphasis throughout is on prevention, which is as it should be. Consequently, geographical distribution is the prominent theme. It is particularly gratifying to see the emphasis on regional distribution of tumours *within* countries, such as that of nasopharyngeal tumours in Japan. The relative frequencies of different types of cancer from many countries from which incidence rates are not yet available are of very great interest. The contrasts between different areas of Indonesia with its 3000 islands are fascinating, as are the relative frequencies of tumours in Java, where oesophageal and stomach tumours together account for only 0·5% compared with cervix 34% (of women's cancer) and liver 5·2%. Figures from countries such as Bangladesh and the Philippines will be new to many.

The space given to specific types of cancer is naturally different from that allocated in western treatises, with stomach heading the list (5 chapters) followed by oral and liver cancer with 3 chapters each.

The screening programmes, diagnostic procedures and criteria, and treatment of stomach cancer in Japan comprise a masterpiece of careful and detailed study, and the 55% 5-year survival rates after curative resection, with 87% in Stage I tumours, deserve high praise.

Relative frequencies of different cancers in sub-groups in India obviously contain vital clues to the causation of cervical and of other forms of cancer. The former has been shown to be 3 times commoner in girls marrying under the age of 14 than in those marrying over the age of 20, and mothers with 9 or more children have 21 times more cervical cancer than nullipara.

No subject is more profusely documented than the relationship between liver cancer and cirrhosis in Japan, so that ample assistance is provided for those studying this unsettled problem.

One most attractive feature is the setting out of all information in short paragraphs, with descriptive headings facilitating reference, and another is the clear, simple and concise diagrams.

This book will be invaluable and interesting to all those in cancer research. It in no sense replaces, but is an adjunct to other works. Its only major drawback is the price, which will deter many.

D. P. BURKITT